# Arterial Spin Labeling Cerebral Perfusion Changes in Chronic Tinnitus With Tension-Type Headache

**DOI:** 10.3389/fneur.2021.698539

**Published:** 2021-08-26

**Authors:** Zhen-Gui Xu, Jin-Jing Xu, Jinghua Hu, Yuanqing Wu, Dan Wang

**Affiliations:** ^1^Department of Otolaryngology, Nanjing Pukou Central Hospital, Pukou Branch Hospital of Jiangsu Province Hospital, Nanjing, China; ^2^Department of Otolaryngology, Nanjing First Hospital, Nanjing Medical University, Nanjing, China

**Keywords:** headache, cerebral blood flow, arterial spin labeling, tinnitus, functional magnetic resonance imaging

## Abstract

**Purpose:** Tinnitus is along with tension-type headache that will influence the cerebral blood flow (CBF) and accelerate the tinnitus severity. However, the potential associations between tension-type headache and tinnitus is still unknown. The current study will explore whether abnormal CBF exists in tinnitus patients and examine the effects of headache on CBF in tinnitus patients.

**Materials and Methods:** Resting-state perfusion magnetic resonance imaging was performed in 40 chronic tinnitus patients and 50 healthy controls using pseudocontinuous arterial spin labeling. Regions with CBF differences between tinnitus patients and healthy controls were investigated. The effects of headache on tinnitus for CBF changes were further explored. Correlation analyses revealed the relationship between CBF values and tinnitus distress as well as CBF values and headache degree.

**Results:** Relative to healthy controls, chronic tinnitus showed decreased CBF, mainly in right superior temporal gyrus (STG), left middle frontal gyrus (MFG), and left superior frontal gyrus (SFG); the CBF in the right STG and the left MFG was negatively correlated with THQ scores (*r* = −0.553, *p* = 0.001; *r* = −0.399, *p* = 0.017). We also observed a significant effect of headache on tinnitus for CBF in the right STG. Furthermore, the headache degree was correlated positively with tinnitus distress (*r* = 0.594, *p* = 0.020).

**Conclusion:** Decreased CBF in auditory and prefrontal cortex was observed in chronic tinnitus patients. Headache may accelerate CBF reductions in tinnitus, which may form the basis for the neurological mechanism in chronic tinnitus with tension-type headache.

## Introduction

Tinnitus, defined as perception of sounds in the absence of external stimuli, has become a pervasive public health issue frequently accompanied with hearing loss, hyperacusis, and related anxious and depressive symptoms (1, 2). Approximately 12–30% of the general population experiences tinnitus worldwide, and consequently brings great social and economic burden to the country (3, 4). Furthermore, almost 26–47% of tinnitus patients also accompanied with headache (5). The relationship between tinnitus and headache has been reported in previous studies (5–7). Thus, headache may be a risk factor that plays a critical role for tinnitus-related impairment. Nevertheless, the potential associations between tinnitus and headache remains elusive.

Prior functional magnetic resonance imaging (fMRI) studies have found that chronic tinnitus is linked with abnormal brain activity in the auditory cortex and non-auditory brain regions (8). Furthermore, tinnitus patients exhibited decreased or increased cerebral blood flow (CBF) in widespread brain regions, including auditory cortex, prefrontal cortex, and parahippocampal gyrus, using single-photon emission computed tomography (SPECT) and positron emission tomography (PET) (9–11). Arterial spin labeling (ASL) could serve as a marker of functional activation, which achieves a direct measure of regional CBF independent of complicated calculations (12). ASL has been used to detect the CBF alterations in neurological or psychiatry disorders (13). However, previous studies did not find any significant CBF changes between tinnitus patients and healthy controls using ASL (14). Regarding headache, previous studies showed that patients with headache showed hypoperfusion, hyperperfusion, or no differences in brain gray matter (GM) (15–17). Further research is required to detect the regions revealing altered CBF in tinnitus and to investigate whether headache may facilitate CBF abnormalities in these regions.

According to the aforementioned findings, we hypothesized that tinnitus might cause abnormal CBF compared to healthy controls and headache might have a potential effect on CBF values in tinnitus patients. The purpose of this study is to evaluate CBF differences between tinnitus patients and healthy controls using the ASL technique and observe the impact of headache on CBF alterations in chronic tinnitus.

## Materials and Methods

### Participants

Forty chronic tinnitus patients and 50 age-, gender-, and education-matched healthy controls were recruited in Nanjing First Hospital (right-handed and at least 9 years of education). There were 15 left-sided and 15 right-sided tinnitus patients as well as 10 tinnitus patients who experienced bilateral tinnitus or tinnitus originating within the head. According to the International Classification of Headache Disorders, Third Edition (beta version) (18), the headache type in our study belongs to tension-type headache. Chronic tinnitus patients were divided into two groups (15 with headache and 25 without headache). The Iowa version of the Tinnitus Handicap Questionnaires (THQ) (19) and pure tone audiometry (PTA) were used to assess tinnitus severity and hearing threshold. All participants had no hearing loss in any of six measured audiometric frequencies ranging from 250 Hz to 8 kHz (hearing thresholds <25 dB). The Hyperacusis Questionnaire was applied to exclude patients with hyperacusis according to the study of Khalfa (20). Participants were excluded if they suffered from pulsatile tinnitus or Meniere's diseases (21), or if they had a past history of migraine, stroke, Alzheimer's disease, Parkinson's disease, brain injury, epilepsy, major depression, sleep disorders (insomnia, obstructive sleep apnea syndrome), MRI contraindications, severe heart diseases, cancer, and damaged liver/kidney function. Patients were not taking medications for treating tinnitus or headache. The Institutional Review Board of Nanjing First Hospital approved the current study. Written informed consent was obtained from all participants before they participated in the study protocol.

The headache intensity and degree were, respectively, measured by the visual analog scale (VAS) and the Headache Impact Test-6 (HIT-6). The status of depression and anxiety were assessed by the Self-Rating Depression Scale (SDS) and the Self-Rating Anxiety Scale (SAS). Clinical and demographic data of the tinnitus patients and healthy controls were shown in Table 1.

**Table 1:** Clinical and demographic data of tinnitus patients and controls.

	**Tinnitus patients (*n* = 40)**	**Controls (*n* = 50)**	***P*-value**
Age (year)	49.05 ± 11.82	47.36 ± 12.39	0.513
Male/Female	15/25	20/30	0.809
Education (years)	12.80 ± 2.92	12.84 ± 3.13	0.951
Duration (months)	37.80 ± 37.07	–	–
THQ score	51.34 ± 13.16	–	–
^a^HIT-6 score	62.49 ± 2.73	–	–
^a^VAS score	5.87 ± 0.84	–	–
SAS score	40.83 ± 6.02	38.92 ± 5.87	0.134
SDS score	41.80 ± 5.79	40.72 ± 4.81	0.336
PTA (left)	16.07 ± 2.53	16.95 ± 2.49	0.099
PTA (right)	16.63 ± 3.12	17.00 ± 2.34	0.517
PTA (average)	16.26 ± 2.59	16.92 ± 1.62	0.131
Gray matter volume (cm^3^)	567.05 ± 29.32	575.52 ± 20.41	0.126
White matter volume (cm^3^)	529.78 ± 20.80	526.58 ± 25.36	0.513
Brain parenchyma volume (cm^3^)	1,096.83 ± 32.76	1,102.10 ± 35.60	0.471

*Data are represented as mean ± SD*.

a *The headache scores were measured for tinnitus patients with headache. PTA, puretone audiometry; THQ, Tinnitus Handicap Questionnaires; HIT-6, Headache Impact Test-6; VAS, Visual Analog Scale; SDS, Self-Rating Depression Scale; SAS, Self-Rating Anxiety Scale*.

### MRI Data

A 3.0 Tesla MRI scanner (Ingenia, Philips Medical Systems, Netherlands) with an eight-channel head coil was used for this study. Functional images were obtained axially using a gradient echo-planar imaging sequence as follows: (a) The resting-state perfusion imaging was performed using a pseudocontinuous arterial spin labeling (pCASL) sequence [repetition time (TR) = 4,000 ms; echo time (TE) =11 ms; label duration = 1,650 ms; post-label delay = 2,000 ms; flip angle (FA) = 90°; field of view (FOV) = 220 mm × 220 mm; slice thickness = 4 mm with 10% gap; matrix = 64 × 64; 20 axial slices; total scan duration = 4 min 18 s]. Finally, each subject contained 60 volumes used as 30 label-control image pairs; (b) Sagittal 3D T1-weighted images were acquired using a three-dimensional turbo fast echo (3D-TFE) T1WI sequence (TR = 8.1 ms; TE =3.7 ms; FA = 8°; FOV = 256 mm × 256 mm; matrix =256 × 256; slice thickness = 1 mm, gap = 0 mm; and 170 sagittal slices; total scan duration = 5 min 29 s).

### Structural Data

Voxel-based morphometry (VBM) approach was applied to calculate the whole brain volumes using the VBM8 toolbox (http://dbm.neuro.uni-jena.de/vbm). DARTEL was used to improve the inter-subject registration of the structural images. Briefly, cerebral tissues were segmented into GM, white matter (WM), and cerebrospinal fluid (CSF) by a unified segmentation algorithm (22). Then, resulting images were normalized to the MNI template, followed by smoothing using an 8-mm full width at half maximum (FWHM) Gaussian kernel. Finally, the resulting voxel-wise GM volume maps were entered as covariates in ASL data analysis.

### ASL Data

The pCASL data were processed using Statistical Parameter Mapping (SPM, version8, http://www.fil.ion.ucl.ac.uk/spm) and ASL data processing toolbox (ASLtbx, https://cfn.upenn.edu/~zewan). Firstly, ASL images were corrected for head motion. Participants whose translations and rotations more than 2 mm and 2°, respectively, were removed from this study. Secondly, CBF maps were calculated using ASLtbx, each participant's CBF map was coregistered to their structural image, and individual structural images were normalized in Montreal Neurological Institute (MNI) space; spatial transforms were concatenated to bring the CBF image to MNI template, with resampling to a 2 mm^3^ × 2 mm^3^ × 2 mm^3^ voxel size. Thirdly, the normalized CBF maps were then spatially smoothed with a Gaussian of 8 mm × 8 mm × 8 mm full-width at half maximum (FWHM). Finally, normalization was performed by dividing the CBF per voxel by the average CBF across the entire brain (23).

### Statistical Analysis

In order to check the normality of clinical and demographic data distribution, the Kolmogorov–Smirnov test was performed. One-way analysis of variance (ANCOVA) and the chi-squared (χ^2^) test was used to investigate the differences among groups. The continuous variables which were not normally distributed were analyzed by Kruskal–Wallis test among groups or by Mann–Whitney *U*-test. SPSS 22.0 software (version 22.0, SPSS Inc., Chicago, IL) was used in above statistical analysis. p < 0.05 was considered to be statistically significant.

Between-group differences in CBF were also analyzed using one-way ANOVA with age, sex, education, and GM volume as nuisance covariates. Significant thresholds were corrected using a false discovery rate (FDR) criterion and set at *p* < 0.01. A full-factorial model was performed to detect the interaction effects of tinnitus and headache on CBF differences. Full factorial analysis was utilized to analyze the main effect and interaction effect of tinnitus and headache. Specifically, the between-subject factors included tinnitus group and headache group. Significant thresholds were corrected using cluster-level family-wise error (FWE), and the threshold was set at *p* < 0.01.

The correlations between aberrant CBF values and each tinnitus characteristic were further investigated. Brain regions showing significant differences between groups were firstly extracted. Then the mean z-values of aberrant CBF mask were computed within each subject. Pearson analysis between the mean z-values and each characteristic were conducted using the SPSS software. Partial correlations were analyzed with age, gender, education, GM volume, and mean hearing levels as covariates. *p* < 0.05 was considered statistically significant.

## Results

### Structural Analysis

No significant differences were detected in the brain GM volume, WM volume and parenchyma volume between chronic tinnitus and healthy controls (Table 1). In addition, we also observed no significant differences of whole-brain volumes between tinnitus patients with headache and without headache.

### CBF Analysis

**Figure 1:**
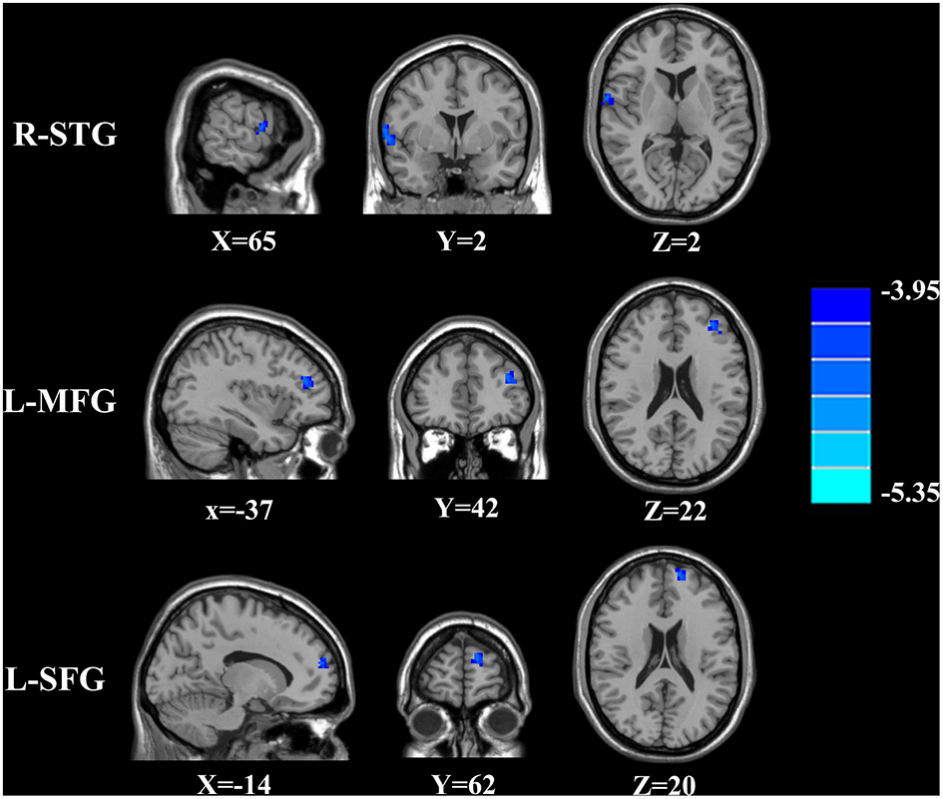
The CBF differences between the chronic tinnitus patients and controls. The tinnitus patients showed reduced CBF in right superior temporal gyrus (STG), left middle frontal gyrus (MFG) and left superior frontal gyrus (SFG) (FWE correction, *p* < 0.01).

**Figure 2:**
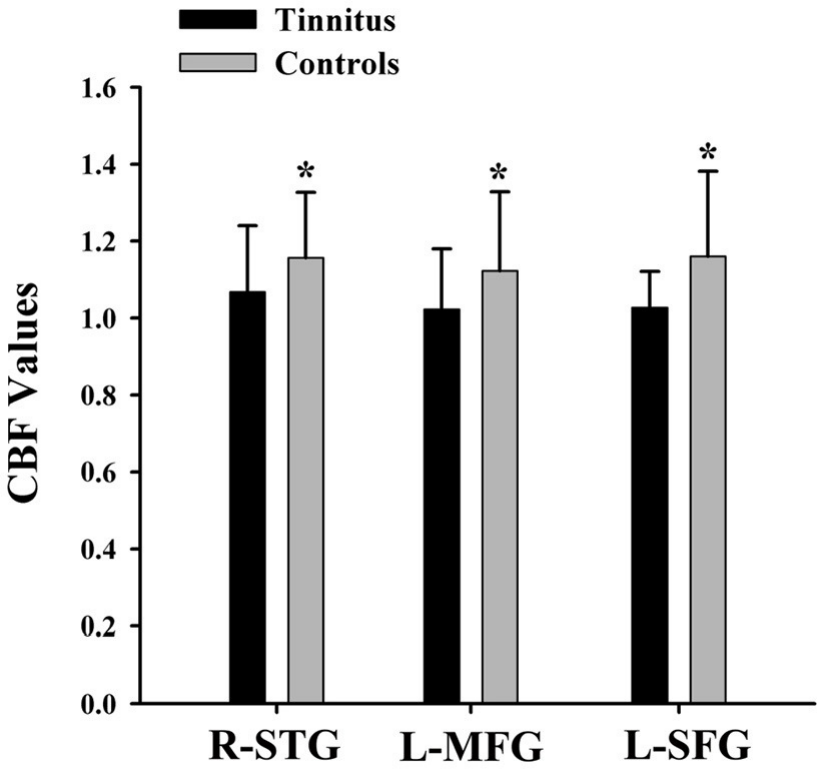
The CBF values of the chronic tinnitus patients and non-tinnitus controls in the right STG (1.07 ± 0.17 vs. 1.16 ± 0.17), left MFG (1.02 ± 0.16 vs. 1.12 ± 0.21), right MFG (1.04 ± 0.17 vs. 1.21 ± 0.19), and left SFG (1.03 ± 0.09 vs. 1.16 ± 0.22) (*p* < 0.01). *means significant differences of CBF values between two groups.

**Figure 3:**
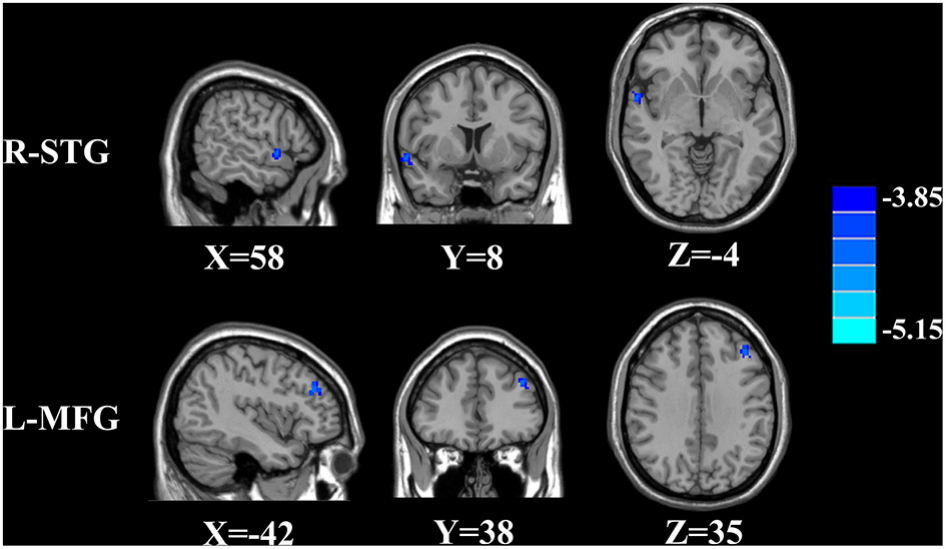
Significant brain regions showing interaction effects on CBF between tinnitus and headache in the right STG and left MFG (FWE correction, *p* < 0.01).

The CBF differences between chronic tinnitus and healthy controls were presented in Figure 1; Table 2. Tinnitus patients revealed reduced CBF in the right superior temporal gyrus (STG), left middle frontal gyrus (MFG), and left superior frontal gyrus (SFG) (*p* < 0.01, FWE corrected). The CBF values for each group are shown in Figure 2. The interaction effect of headache on tinnitus was significant in the right STG and left MFG (Figure 3; Table 3) (*p* < 0.01, FWE corrected). When the tinnitus patients suffered from a headache symptom, the CBF would be decreased.

**Table 2:** Significant CBF differences between tinnitus patients and controls.

**Brain regions**	**Brodmann area**	**Peak MNI coordinates x, y, z (mm)**	**Peak value**	**Voxels**
R-STG	22	65, 2, 2	−4.3156	162
L-MFG	10	−37, 42, 22	−4.5847	205
L-SFG	11	−14, 62, 20	−4.5682	232

*These clusters are referred to multiple comparisons correction using the FWE rate (p < 0.01). STG, superior temporal gyrus; MFG, middle frontal gyrus; SFG, superior frontal gyrus*.

**Table 3:** Significant brain regions showing interaction effect on CBF between tinnitus and headache.

**Brain regions**	**Brodmann area**	**Peak MNI coordinates x, y, z (mm)**	**Peak value**	**Voxels**
R-STG	22	58, 8, −4	−4.0259	163
L-MFG	10	−42, 38, 35	−4.2350	122

*These clusters are referred to multiple comparisons correction using the FWE rate (p < 0.01). STG, superior temporal gyrus; MFG, middle frontal gyrus*.

### Correlation Analysis

**Figure 4:**
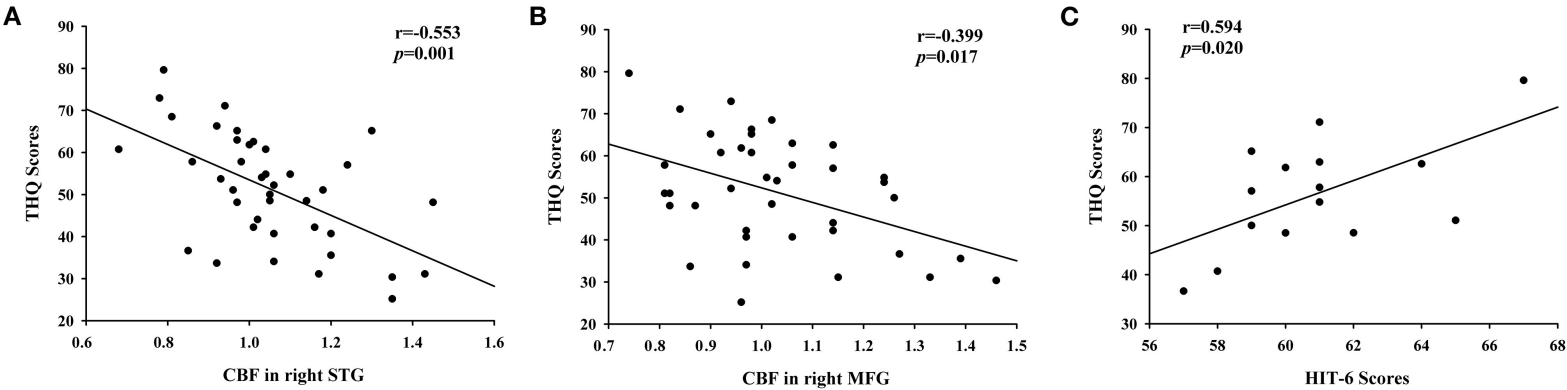
The correlations between the CBF alterations and tinnitus characteristics. **(A)** CBF in right STG was negatively correlated with THQ scores (*r* = −0.553, *p* = 0.001); **(B)** CBF in right MFG was negatively correlated with THQ scores (*r* = −0.399, *p* = 0.017); **(C)** The HIT-6 scores were positively associated with the THQ scores (*r* = 0.594, *p* = 0.020).

The significant correlations between the CBF differences and clinical data are shown in Figure 4. The CBF in the right STG and the left MFG was negatively correlated with THQ scores (*r* = −0.553, *p* = 0.001; *r* = −0.399, *p* = 0.017). Regarding the correlations between headache degree and tinnitus severity, the HIT-6 scores were positively correlated with the THQ scores (*r* = 0.594, *p* = 0.020). However, the VAS scores were not significantly correlated with the THQ scores (*p* > 0.05).

## Discussion

The current study explored for the first time the associations between tension-type headache and tinnitus using the ASL technique. Our current study revealed greater cerebral perfusion impairment for tinnitus patients suffering from comorbid headaches and suggested an interrelation between tinnitus and headache syndromes. The observed increased impairment in tinnitus patients with headache can be explained as an additive effect of both disorders on health-related quality of life (6). The more frequent occurrence of further comorbidities suggests a generally increased amplification of sensory signals in a subset of tinnitus patients with comorbid headache. Previous studies also found a highly significant association between tinnitus laterality and headache laterality (5). An even higher correlation might be obtained by asking explicitly for side changes of headaches and tinnitus.

Our tinnitus patients showed reduced CBF in the right STG that was correlated with THQ scores. Dysfunction of the temporal cortex is associated with affective disturbance, which is associated with the headache symptom (24, 25). Tinnitus comorbid with headache may lead to more complex dysfunction in the cortico-limbic network. Moreover, prior fMRI researches have revealed the associations between aberrant brain activity or connectivity of the STG and tinnitus severity (26, 27). However, prior studies using SPECT or PET did not detect any reduced cerebral perfusion in the auditory cortex (9–11), which was different from our current results. Different sample sizes and imaging techniques may contribute to this discrepancy. The prefrontal cortex plays a pivotal role in emotional processing and executive function (28). Disrupted neural activity was found in the executive attention network, including the MFG and SFG (8). Previous fMRI studies have also pointed out that the abnormalities of the prefrontal cortex could act as a direct mechanism in tinnitus chronification (29, 30). Our results indicated that CBF alterations in the prefrontal cortex may become important brain characteristics for tinnitus. Nonetheless, the clinical implication for decreased CBF in the prefrontal cortex remains unknown and needs to be further explored.

Prior studies have confirmed that headache patients showed decreased CBF in temporal and prefrontal cortex (31, 32). Our findings indicate that the headache aggravates the CBF decreases in tinnitus patients, leading to attention and executive dysfunction. Moreover, the headache degree is positively associated with tinnitus severity, which was in accordance with prior researches (31–33). However, the relationship between headache and chronic tinnitus has not been substantially elucidated and still requires to be confirmed in the future.

Several limitations have to be acknowledged in this study. Firstly, the relatively small sample size in the current investigation might restrict the statistical power. Future study with a larger data set is warranted. Secondly, we just examined the headache symptom by using HIT-6 and VAS scores. More neurocognitive tests are required to detect the headache characteristic, such as the Migraine Disability Assessment Questionnaire (MIDAS) (34) and Visual Light Sensitivity Questionnaire-8 (VLSQ-8) (35). Furthermore, we did not compare the CBF differences between those who have tinnitus and those who do not in headache subjects in order to see the effects of tinnitus. We speculate that tinnitus can cause not only the CBF abnormalities but the changes of neuronal activity in headache patients, but this requires to be confirmed in a further study. Finally, only the CBF in each brain region was measured but we did not analyze the CBF connectivity among different brain regions. The whole-brain CBF connectivity analysis needs to be considered in further study.

In conclusion, our findings suggest that CBF abnormalities may contribute to the neuropathological mechanisms underlying chronic tinnitus with headache. The current study identified a history of headache as a clinical risk factor for the development of tinnitus. Therefore, clinicians should pay close attention to the history of headache when they care about patients with tinnitus comorbid with hearing disease.

## Data Availability Statement

The raw data supporting the conclusions of this article will be made available by the authors, without undue reservation.

## Ethics Statement

The studies involving human participants were reviewed and approved by the Research Ethics Committee of the Nanjing Medical University prior to study initiation. The patients/participants provided their written informed consent to participate in this study.

## Author Contributions

Z-GX and J-JX designed the experiment, collected the data, performed the analysis, and wrote the manuscript. JH helped collect the data and perform the analysis. YW and DW contributed to the discussion and manuscript revision. All authors contributed to the article and approved the submitted version.

## Conflict of Interest

The authors declare that the research was conducted in the absence of any commercial or financial relationships that could be construed as a potential conflict of interest.

## Publisher's Note

All claims expressed in this article are solely those of the authors and do not necessarily represent those of their affiliated organizations, or those of the publisher, the editors and the reviewers. Any product that may be evaluated in this article, or claim that may be made by its manufacturer, is not guaranteed or endorsed by the publisher.
